# The Role of 3-Dimensional Power Doppler Imaging in the Assessment of Ovarian Teratoma in Pregnancy: A Case Report

**DOI:** 10.1155/2011/896396

**Published:** 2011-08-25

**Authors:** Konstantinos Kalmantis, Christos Iavazzo, Vasiliki Anastasiadou, Aris Antsaklis

**Affiliations:** Department of Obstetrics and Gynecology, IASO Hospital, 10433 Athens, Greece

## Abstract

*Background*. Conventional sonography is the primary imaging tool for these pregnant women who present with an ovarian teratoma. In some cases, however, sonography diagnosis is difficult. We report a case of ovarian teratoma during pregnancy diagnosed by three-dimensional Power Doppler. The cyst was removed via laparotomy without fetal or maternal complications. Three-dimensional ultrasound with multiplanar view can better discriminate a benign ovarian teratoma from complex ovarian lesions or malignant tumors. Its role is significant especially during pregnancy as it may assist in determining which patients are requiring surgery and which are not. The results of three-dimensional sonography and magnetic resonance (MR) were equal but the role of MR imaging is limited in early pregnancy. *Conclusions*. Three-dimensional technique is a reliable diagnostic modality for preoperative assessment of an ovarian teratoma as it can be performed during the first trimester of pregnancy.

## 1. Introduction

The incidence given for ovarian tumors complicating pregnancy varies in the literature [1–3]. During the first trimester, the most common pelvic mass is the corpus luteum cyst, which usually regresses by the 10th to 15th gestational week [4]. Most other ovarian masses discovered during pregnancy are benign dermoids cysts which account for 20%–40% of ovarian neoplasms [1, 2, 5]. Complication associated with dermoid cysts may include torsion, rupture, and malignant transformation. Moreover, dermoid cysts may complicate labor by obstructing the birth canal. Surgery is usually performed during pregnancy because 2%–5% of these masses are malignant and even benign masses can cause obstetric complications [6]. Pregnancy is a special state in which the rate of complications such as torsion and rupture may increase because of the increasing size of uterus.

The sonographic appearance of dermoid cysts can range from a purely cystic to a complex to a purely solid mass. Conventional sonography can easily identify a dermoid cyst. However, those that contain hair, sebum, and calcium can produce acoustical shadowing which hides most of the mass and makes the sonographic diagnosis difficult [7]. Color Doppler can serve as a useful adjunct to B-mode assessment for detecting adnexal malignant lesions in a gravid population, however, the limitations of velocimentry should be recognized. The sensitivity of color Doppler sonography is tempered by the considerable overlap in blood flow patterns, causing frequent incorrect assignment of malignant potential [8].

Three-dimensional technique is a new emerging technology that provides additional information for the evaluation of ovarian tumors. Multiplanar view of the dermoid tumor allows visualization of the intratumoral calcification or identification of the bones structures. This technique can better evaluate complex ovarian tumors such as dermoids, which may give a wrong impression of malignancy when using conventional sonography.

The aim of our study is to present the diagnosis by three-dimensional Power Doppler and management of a case of a pregnant woman with an ovarian dermoid cyst.

## 2. Case Report

**Figure 1:**
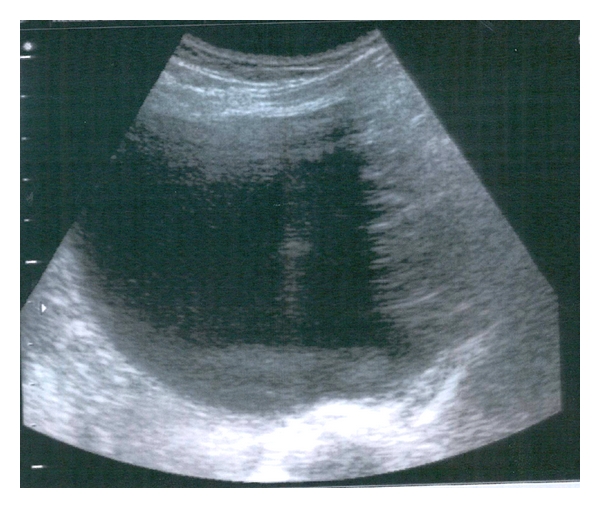
A cyst in the left ovary with mixed echogenicity and irregular inner contents.

**Figure 2:**
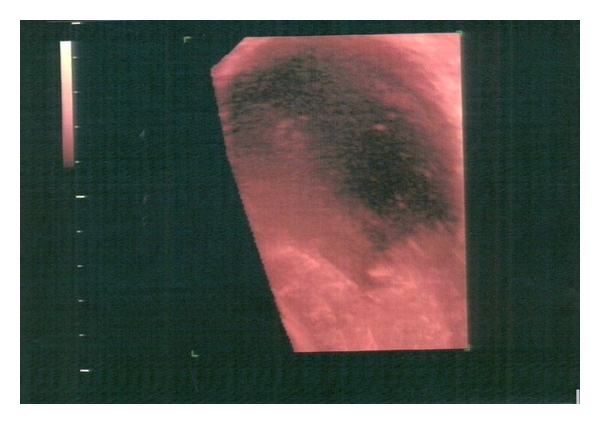
Three-dimensional image of a dermoid cyst. The hyperechoic solid part and speckled appearance of the sebaceous contents are seen with the use of minimum maximum mode.

**Figure 3:**
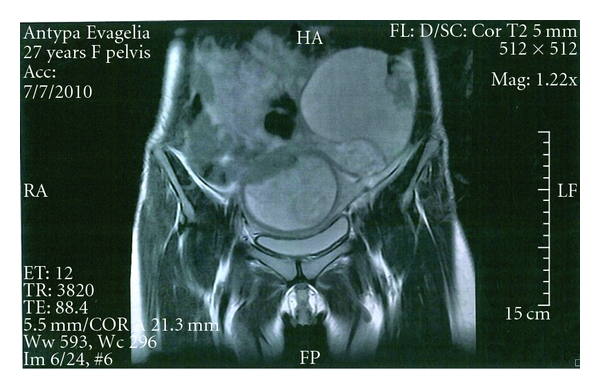
MRI of a mature cystic teratoma of the left ovary. A large cystic mass with component of high sigual intensity, consistent with fat.

An ultrasound scan was performed at 8th gestational week in a 28-year-old primigravida which revealed an 8 × 6 × 7 cm smooth-walled, mixed echogenic mass with irregular inner contents in the left ovary (Figure 1) and a viable singleton intrauterine pregnancy. A repeat scan four weeks later, showed an increase in the size of the mass. Its largest diameter was 9.85 cm and three-dimensional Power Doppler with the use of minimum-maximum mode revealed a hyperechoic solid part and speckled appearance of sebaceous contents suggesting a dermoid cyst (Figure 2). Power Doppler didnot detect any vascularization neither at the periphery nor in the central region of the cyst. A magnetic resonance at 15th gestational week imaging MRI showed signal intensity consistent with fat allowing characterization of the mass as a mature cystic teratoma (Figure 3). Tumor marker Ca_125_ was negative (31 u/mL). Differential diagnosis included serous and mucinous cystadenoma, endometrioma, and malignant tumors such as mucinous cystadenocarcinoma, serous cystadenocarcinoma, and embryonal carcinoma. Following extensive discussion about the risks and benefits the patient opted for surgical removal. It was decided to proceed with exploratory laparotomy at 16 weeks gestation under general anesthesia with resection of the left adnexal mass and preservation of as much ovarian tissue as possible. The fetus tolerated the operation well. The final pathological diagnosis was “mature cystic teratoma.”

## 3. Discussion

Sonographic evaluation of a pregnant woman with a pelvic mass presents a unique diagnostic problem. Conventional sonography is the primary imaging tool in pregnant women who present with an ovarian dermoid cyst. Although dermoid cyst has a diagnostic appearance, in some cases, it can range from a purely cystic to a complex to a purely solid mass. In these cases, MRI will increase diagnostic accuracy because of high sensitivity for fat contents [9, 10].

Benign cystic teratoma is the most common ovarian neoplasm diagnosed in pregnancy [2]. Treatment is surgical removal as soon as possible after diagnosis. The sonographic appearance of ovarian teratoma may be entirely echo-free or may have a few septa while the complex type has varying degrees of cystic and solid elements. Hair, sebum, calcifications, teeth, and bone may promote acoustic shadow. These sonographic findings widen the differential diagnosis dramatically to include multiple benign entitles (corpus lutem cyst, pedunculated calcified fibroid, bowel gas, and endometriosis) with the occasional malignant lesion [11]. However two-dimensional ultrasonography is not able in some cases to distinguish cystic teratoma, preoperatively, from other benign tumors and to rule out malignancy especially in pregnancy where the mobility and position of the tumor may change due to enlarged uterus. For this reason, two-dimensional Power Doppler examination could be used to examine the color content of the solid parts of this tumor. This information combined with the gray-scale assessment and MRI—although some would argue against it—would be potentially useful for cancer detection or exclusion.

Three-dimensional ultrasound provides an improved echogenicity of the ovarian anatomy and can better discriminate a dermoid cyst from complex ovarian lesions or malignant tumors. Especially in pregnancy it may assist in determining which patients are requiring surgery as opposed to those patients with other benign ovarian masses (corpus luteum cysts and endometrioma) who can be followed.

The addition of the three-dimensional ultrasound allowed better visualization of the inner wall irregularities, the wall thickness, the presence of thick septations or solid areas, evaluation of the echogenicity of the lesion and analysis of the distal shadowing [12]. Multiple sections of the tumor, rotation, translation, and reconstruction of three-dimensional plastic images allowed more precise evaluation of the tumor, without increasing the scanning time or patients' discomfort [9].

MRI in pregnant women is particularly valuable when two-dimensional sonography is unable to show whether a pelvic mass represents a fibroid or an ovarian lesion since a confident diagnosis of a uterine fibroid may eliminate the need for surgery during pregnancy [13]. Demonstration of fat on MRI allows characterization at the lesion as a mature cystic teratoma and confirmation of three-dimensional ultrasonographic findings. Umesaki et al. reported a case of dermoid cyst with fat balls in which three-dimensional imaging easily detected them and showed almost the same findings that were observed macroscopically after surgery. MRI clearly revealed the character of floating globules [14]. In women presenting with a pelvic mass, early in pregnancy, MRI, should be avoided for two reasons. Firstly, is too expensive and secondly although there is no evidence to suggest that MRI is hazardous to the embryo at the magnetic field strength and ratio frequency, the first trimester represents the major period of organogenesis. Thus, the British National Radiological Protection board has suggested that “it might be prudent to exclude pregnant women during the first trimester” [15].

Finally, the tumor marker Ca_125_ can be elevated inherent to pregnancy itself, thus, it is primarily used to monitor recurrence of ovarian cancer rather than to establish diagnosis due to lack of specificity [16]. Precise characterization of the pelvic mass is essential to either plan a surgery in the pregnant woman or confidently postpone surgery. Ovarian cystectomies or oophorectomies via laparoscopy or laparotomy should be performed at 14–16 weeks of gestation to avoid the risk of damage to the corpus luteum [10].

In our case we performed three-dimensional ultrasound in order to characterize the ovarian tumor during pregnancy. Three-dimensional ultrasound provides an improved recognicity of the ovarian anatomy and can better discriminate a benign ovarian teratoma from complex ovarian lesions or malignant tumors. In the current literature three-dimensional imaging can enhance and facilitate the morphologic evaluation of both benign and malignant adnexal tumors [17, 18]. The multiplanar view can be helpful for reducing the false positive rate in cystic-solid and solid vascularized adnexal masses and might help to identify women if needed, to have less invasive surgical procedure [19, 20]. However, according to some studies, no clear difference was shown between three-dimensional Power Doppler and conventional imaging [21, 22]. Moreover, Jokubkiene et al. have clearly demonstrated that even though two-dimensional and three-dimensional Power Doppler ultrasound can be used to discriminate between benign and malignant ovarian tumors, their use adds little to a correct diagnosis of malignancy in an ordinary population of ovarian tumors [23]. They believe that objective quantification of the color content of the tumor scan using three-dimensional Power Doppler ultrasound does not seem to add more to gray-scale imaging than does subjective quantification by the ultrasound examiner using two-dimensional Power Doppler ultrasound [23]. Also, one could state that there is no sufficient explanation what are possible advantages of three-dimensional Power Doppler over other more simple methods such as conventional “pattern recognition” or the so called “Risk of Malignancy Index” (RMI) method which use only two-dimensional sonographic imaging [24]. In a recent study, it has been shown that in over 400 tumors examined by three-dimensional Power Doppler and RMI the sensitivity of RMI for prediction of malignancy was 88%, with a cutoff value of 202.5 at 95% confidence interval whereas the sensitivity of three-dimensional Power Doppler for prediction of malignancy was 75% while adding three-dimensional Power Doppler to RMI increased its sensitivity to 99% [24].

In conclusion, three-dimensional imaging is a reliable diagnostic modality, less expensive than MRI for preoperative assessment of a dermoid tumor which can be performed in the first trimester of pregnancy, with no danger for the fetus and without increasing time or woman's discomfort.
